# Supplementary material for: Pain Assessment Using the Analgesia Nociception Index (ANI) in Patients Undergoing General Anesthesia: A Systematic Review and Meta-Analysis
Source: J Pers Med. 2023 Oct 4;13(10):1461. doi: 10.3390/jpm13101461 (PMC10608238; doi:10.3390/jpm13101461)
Supplement: Supplementary file 1 [file jpm-13-01461-s001.zip › jpm-2598805-supplementary.pdf]

Supplementary Materials

Figure S1A. Bivariate SROC curve for the prediction of intraoperative painful stimuli

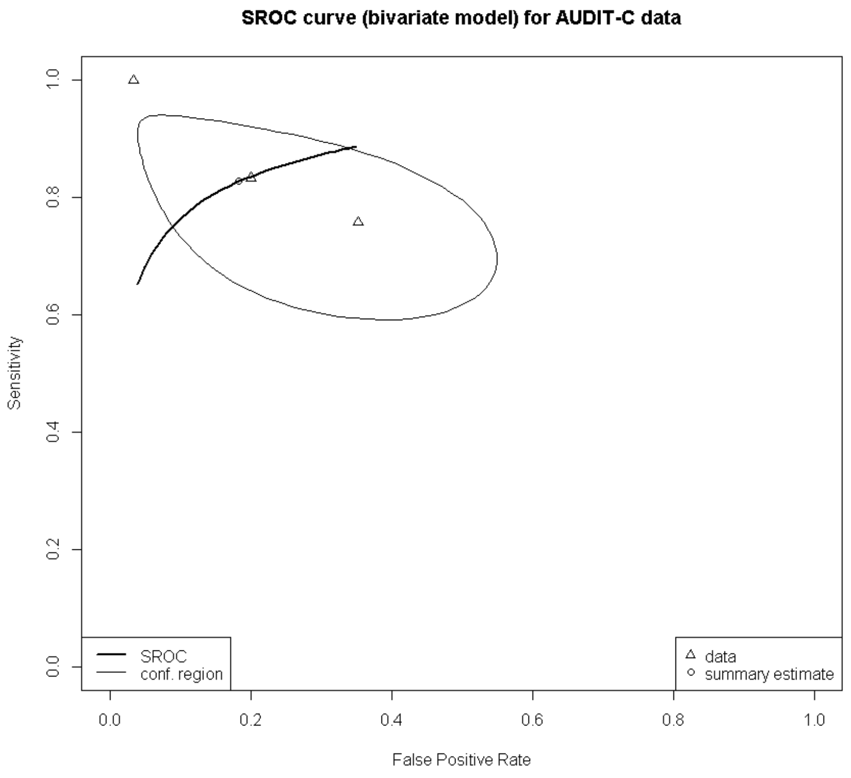

Bivariate SROC curve of ANI for the prediction of intraoperative pain. The triangles indicate each individual study. The summary point (circle) represents the average sensitivity and specificity estimates from the study results, and its corresponding 95% confidence region (gray line) is illustrated. The 95% prediction region represents the confidence region for a forecast of true sensitivity and specificity in a future study.

Figure S1B. Proportional hazard ROC curve for the prediction of intraoperative pain

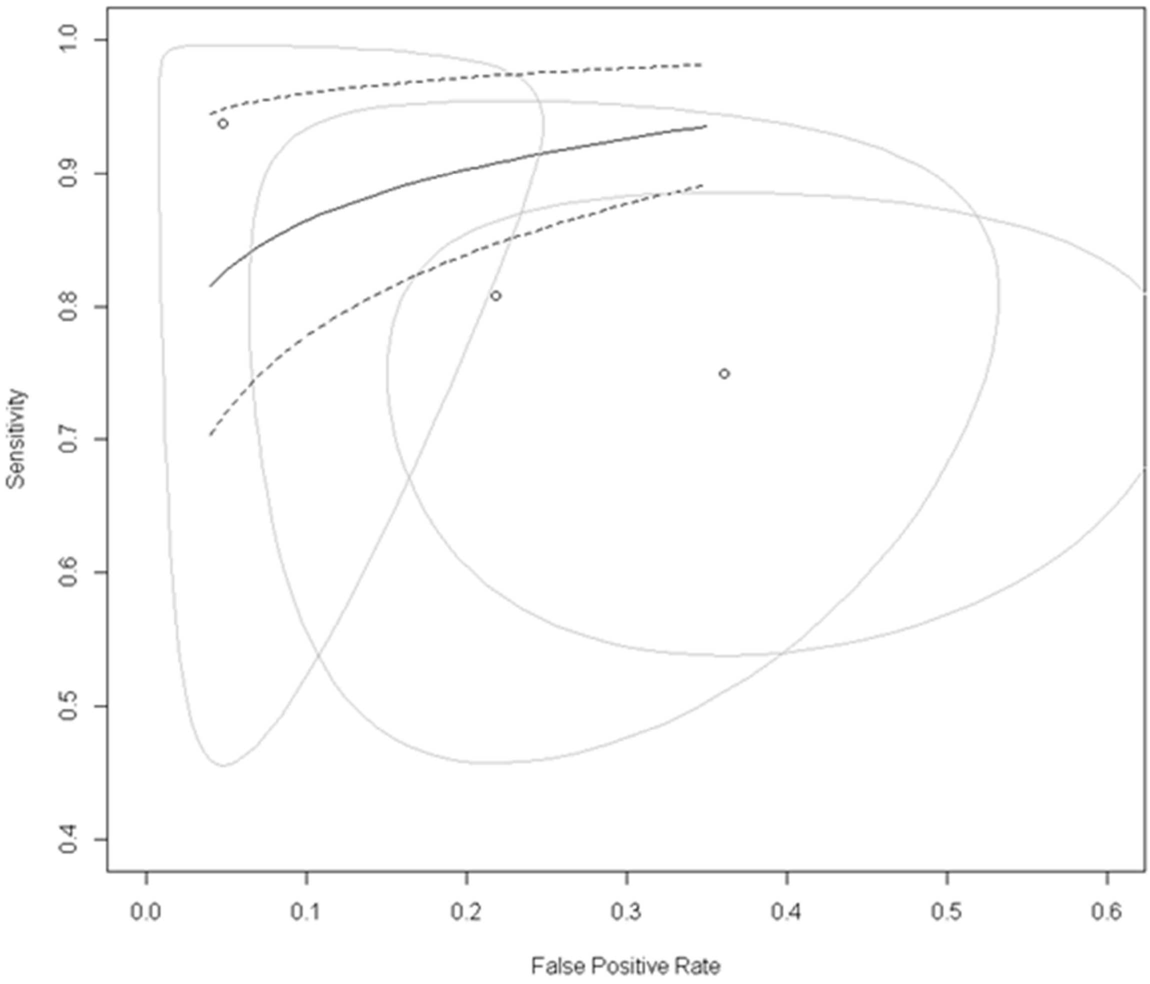

Proportional hazard ROC curve of ANI for the prediction of intraoperative pain. The circles indicate each individual study and corresponding 95% confidence region (thin gray lines). Thick black line represents summary estimate and 05% confidence region (black dashed line). The 95% prediction region represents the confidence region for a forecast of true sensitivity and specificity in a future study.

Figure S2A. Bivariate SROC curve for the prediction of postoperative pain

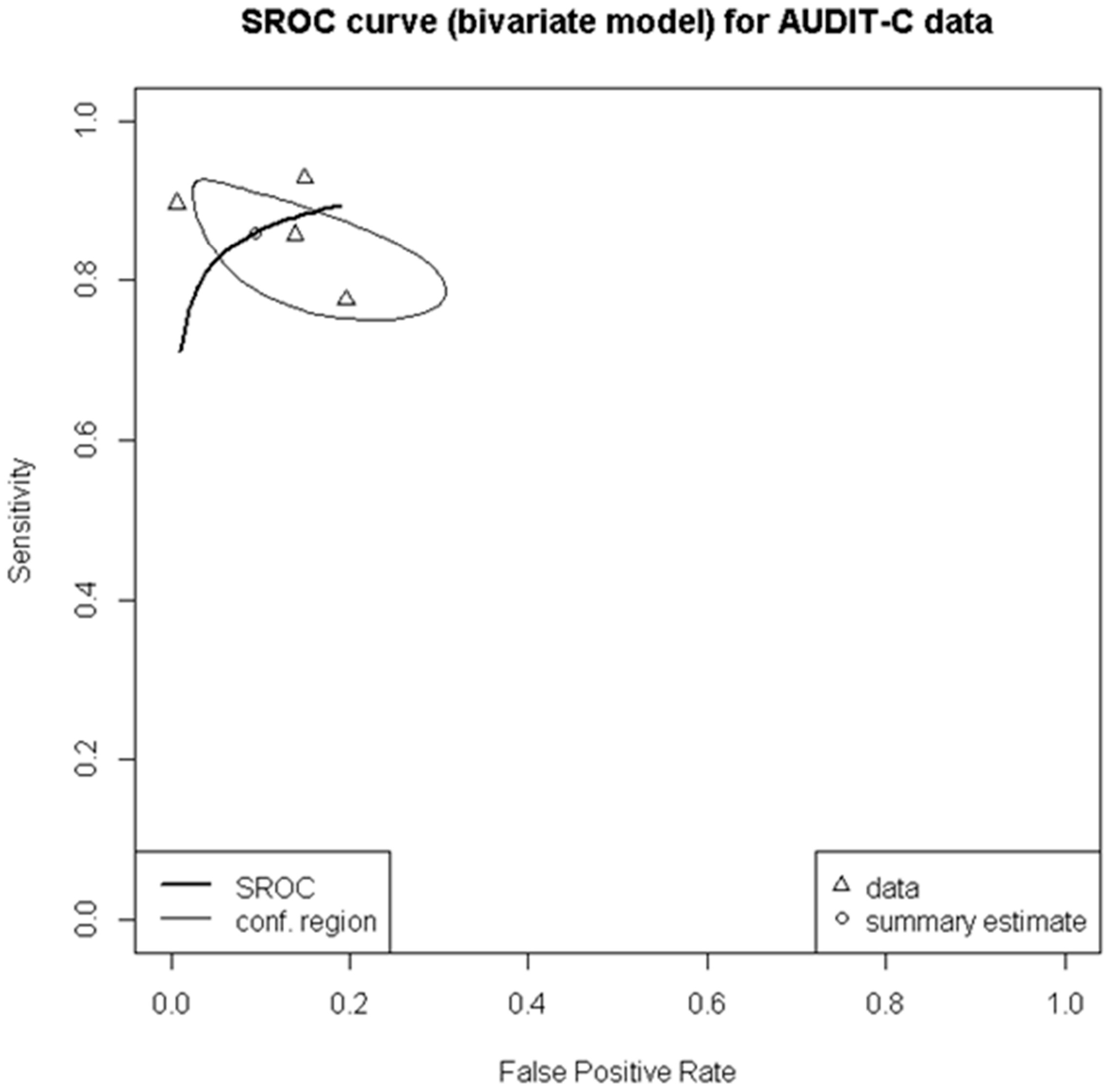

Bivariate SROC curve of ANI for the prediction of postoperative pain. The triangles indicate each individual study. The summary point (circle) represents the average sensitivity and specificity estimates from the study results, and its corresponding 95% confidence region (gray line) is illustrated. The 95% prediction region represents the confidence region for a forecast of true sensitivity and specificity in a future study.

Figure S2B. Proportional hazard ROC curve for the prediction of postoperative pain

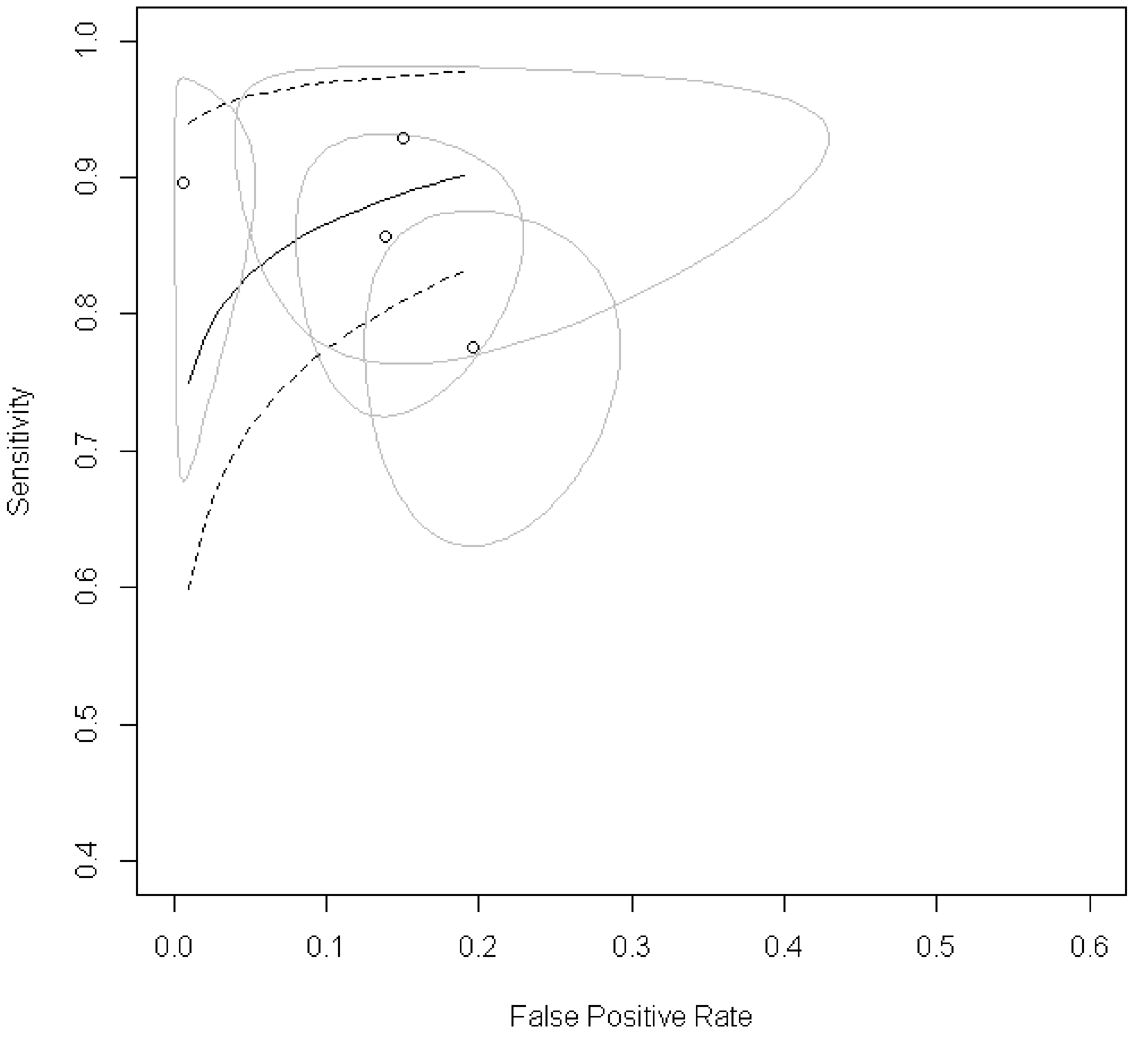

Proportional hazard ROC curve of ANI for the prediction of postoperative pain. The circles indicate each individual study and corresponding 95% confidence region (thin gray lines). Thick black line represents summary estimate and 05% confidence region (black dashed line).The 95% prediction region represents the confidence region for a forecast of true sensitivity and specificity in a future study.

Figure S3. **Forest plot of specificity excluding Charier 2019**

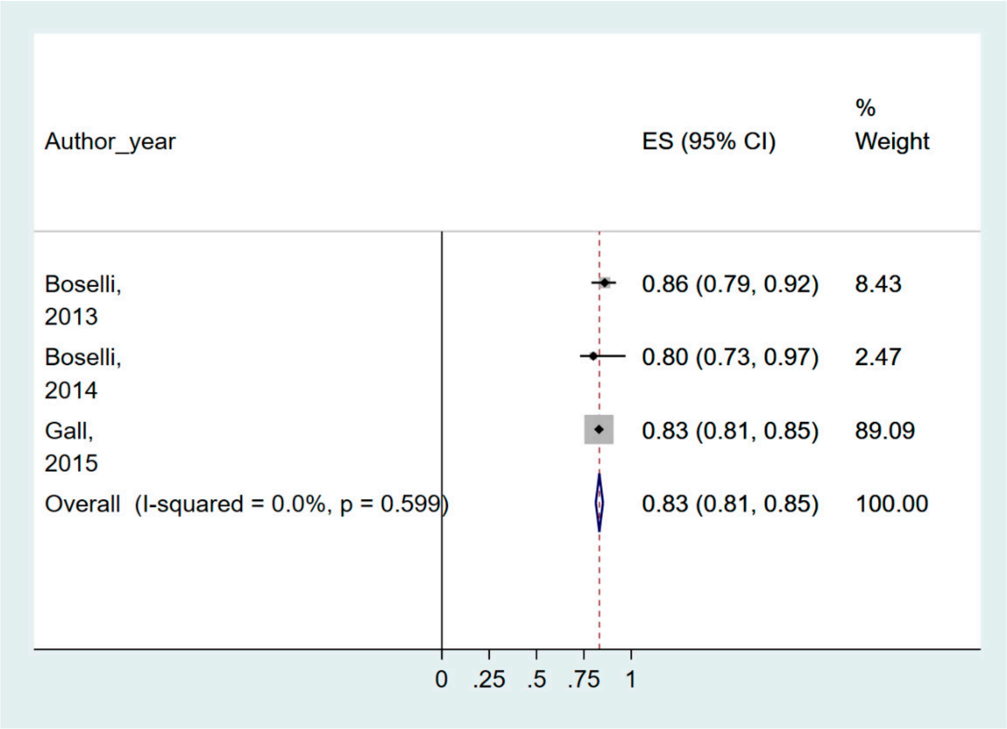

The figure depicts individual trials as filled squares with relative weight and the 95% confidence interval (CI) of the difference as a solid line. The diamond shape indicates the pooled estimate and uncertainty for the combined effect.
